# Genomic characterisation of clinical and environmental *Pseudomonas putida* group strains and determination of their role in the transfer of antimicrobial resistance genes to *Pseudomonas aeruginosa*

**DOI:** 10.1186/s12864-017-4216-2

**Published:** 2017-11-10

**Authors:** Silke Peter, Philipp Oberhettinger, Leonard Schuele, Ariane Dinkelacker, Wichard Vogel, Daniela Dörfel, Daniela Bezdan, Stephan Ossowski, Matthias Marschal, Jan Liese, Matthias Willmann

**Affiliations:** 1 Institute of Medical Microbiology and Hygiene, University of Tübingen, Tübingen, Germany; 2 German Center for Infection Research (DZIF), partner site Tübingen, Tübingen, Germany; 3 Centre for Genomic Regulation (CRG), The Barcelona Institute of Science and Technology, Barcelona, Spain; 4 Medical Center, Department of Hematology, Oncology, Immunology, Rheumatology & Pulmonology, University of Tübingen, Tübingen, Germany; 5 Clinical Collaboration Unit Translational Immunology, German Cancer Consortium (DKTK) and German Cancer Research Center (DKFZ), Partner site Tübingen, Tübingen, Germany; 6 Universitat Pompeu Fabra (UPF), Barcelona, Spain; 7 Department of Medical Microbiology, Universitair Medisch Centrum Groningen, Groningen, Netherlands; 8 Department of Physiology and Biophysics, Weill Cornell Medicine, New York, New York, USA; 9 The HRH Prince Alwaleed Bin Talal Bin Abdulaziz Alsaud Institute for Computational Biomedicine, Weill Cornell Medicine, New York, New York, USA; 10 Institute of Medical Genetics and Applied Genomics, University of Tuebingen, Tübingen, Germany

**Keywords:** *Pseudomonas putida*, VIM, Horizontal gene transfer

## Abstract

**Background:**

*Pseudomonas putida* is a Gram-negative, non-fermenting bacterium frequently encountered in various environmental niches. *P. putida* rarely causes disease in humans, though serious infections and outbreaks have been reported from time to time. Some have suggested that *P. putida* functions as an exchange platform for antibiotic resistance genes (ARG), and thus represents a serious concern in the spread of ARGs to more pathogenic organisms within a hospital. Though poorly understood, the frequency of ARG exchange between *P. putida* and the more virulent *Pseudomonas aeruginosa* and its clinical relevance are particularly important for designing efficient infection control strategies, such as deciding whether high-risk patients colonized with a multidrug resistant but typically low pathogenic *P. putida* strain should be contact isolated or not.

**Results:**

In this study, 21,373 screening samples (stool, rectal and throat swab) were examined to determine the presence of *P. putida* in a high-risk group of haemato-oncology patients during a 28-month period. A total of 89 *P. putida* group strains were isolated from 85 patients, with 41 of 89 (46.1%) strains harbouring the metallo-beta-lactamase gene *bla*_VIM_. These 41 clinical isolates, plus 18 *bla*_*VIM*_ positive environmental *P. putida* isolates, and 17 *bla*_*VIM*_ positive *P. aeruginosa* isolates, were characterized by whole genome sequencing (WGS).

We constructed a maximum-likelihood tree to separate the 59 *bla*_VIM_ positive *P. putida* group strains into eight distinct phylogenetic clusters. *Bla*_VIM-1_ was present in 6 clusters while *bla*_VIM-2_ was detected in 4 clusters. Five *P. putida* group strains contained both, *bla*_VIM-1_ and *bla*_VIM-2_ genes.

In contrast, all *P. aeruginosa* strains belonged to a single genetic cluster and contained the same ARGs. Apart from *bla*_VIM-2_ and *sul* genes, no other ARGs were shared between *P. aeruginosa* and *P. putida*. Furthermore, the *bla*_VIM-2_ gene in *P. aeruginosa* was predicted to be only chromosomally located.

**Conclusion:**

These data provide evidence that no exchange of comprehensive ARG harbouring mobile genetic elements had occurred between *P. aeruginosa* and *P. putida group* strains during the study period, thus eliminating the need to implement enhanced infection control measures for high-risk patients colonized with a *bla*_VIM_ positiv *P. putida* group strains in our clinical setting.

**Electronic supplementary material:**

The online version of this article (10.1186/s12864-017-4216-2) contains supplementary material, which is available to authorized users.

## Background

*Pseudomonas putida* is a Gram-negative, rod-shaped, non-fermenting bacterium that is ubiquitously encountered in the environment. It harbours a broad spectrum of metabolic enzymes, allowing the species to adapt to various niches, including soil and water-associated habitats [1]. *P. putida* is a rare cause of infection in humans. However, there are several reports of bacteraemia, wound and eye infections, urinary tract infection, pneumonia, central venous catheter infection and soft tissue infections caused by *P. putida* [2–9]. A high proportion of *P. putida* bacteraemia is considered to be catheter-related, occurring predominantly in immunocompromised hosts and generally associated with a low rate of mortality [2, 10, 11]. However, lethal cases of bacteraemia and soft skin tissue infections have been described [3, 4]. While the antimicrobial resistance patterns of clinical isolates varied between studies, multidrug-resistant *P. putida* isolates harbouring metallo-β-lactamase (MBL) genes have been reported from all over the world [6–9, 12–19]. MBLs belong to the molecular class B β-lactamases and are characterized by requiring zinc for the catalysis of β-lactams [20]. MBLs can confer resistance to clinically important β-lactams including carbapenems which are often considered as last line of defence in critically ill patients. Aggravating the treatment limitation, the most important enzymes of this group, Verona integron-encoded metallo-β-lactamase (VIM), Imipenemase (IMP), and New Delhi metallo-β-lactamase (NDM), are often co-localized with other resistance conferring genes on mobile genetic elements in *P. aeruginosa* [21].

Several outbreaks of *P. putida* occurred on ICU (intensive care units) as well as non-ICU wards, some of which were related to the transmission of contaminated fluids [22–25]. This underlines the ability of *P. putida* not only to colonize patients, but also to persist in fluids and in water-associated hospital environments [13]. The role of environmental bacteria like Pseudomonas species behaving as reservoirs and vectors of resistance determinants in hospital water systems has become a serious concern to infection control professionals. Investigations into bacteria isolated from hospital wastewater detected *bla*_VIM_ MBL genes in various bacterial species [26]. It was suggested that *P. putida* functions as an exchange platform for genetic elements between environmental and clinical strains. This was evident in the analysis of a *P. putida* carbapenem resistance-conferring conjugative plasmid, which contained genetic regions of both clinical and environmental microbiota [27]. Additionally, the characterization of genetic resistance elements of eight *P. putida* and eleven *P. aeruginosa* clinical isolates suggested horizontal dissemination of *bla*_VIM-2_ in a subset of strains [28]. However, the detailed mechanisms, frequency of antimicrobial resistance exchange and relevance between *P. putida* and *P. aeruginosa* remain poorly understood.

Following an outbreak of multidrug-resistant *P. aeruginosa* strains, weekly active screening cultures (ASC) were introduced for the detection of Pseudomonas species at the haemato-oncology wards in our hospital [29]. Interestingly, *P. putida* was frequently recovered during ASC besides *P. aeruginosa*. In one case, a *bla*_VIM_ positive *P. putida* and a *bla*_VIM_ positive *P. aeruginosa* strain were isolated from a single stool sample, suggesting that the transfer of ARGs might have occurred between the two species in the patient. The dissemination of multidrug resistance elements to successful *P. aeruginosa* clones represents a serious health care concern [28]. This scenario, in combination with the fact that *bla*_VIM_ positive *P. aeruginosa* strains were continuously isolated in our patients made it difficult to establish infection control strategies for patients colonized with *bla*_VIM_ positive *P. putida*. Systematically gathered data were not available to help us form the basis for a recommendation, a point that encouraged us to conduct this study.

In order to characterize the role of *P. putida* group strains in transmitting determinants of antimicrobial resistance to *P. aeruginosa* within the patient and the patient-related environment, an observational study was conducted over a 28-month period. Our aim was to evaluate i) the frequency of colonisation with *P. putida* in a high-risk haemato-oncological population, ii) the antimicrobial susceptibility patterns, iii) the genetic relatedness of *P. putida* and *P. aeruginosa* strains and iv) to compare genetic antibiotic resistance elements from *P. putida* and *P. aeruginosa* strains isolated from the same patient cohort and environment during the study period.

## Methods

### Strains included in the study

A total of 21,373 screening samples obtained from a high-risk group of hemato-oncology patients were examined over a study period of 28 months. The samples were obtained as part of routine care. To address the study objectives, we included the following isolates: i) the first *P. putida* isolate per patient, ii) all *bla*_VIM_ positive *P. aeruginosa* isolates recovered from the same patient cohort during the study period and iii) all *bla*_VIM_ positive *P. putida* and *P. aeruginosa* recovered from patient-associated environmental sources. The detailed workflow of the study is depicted in Additional file 1: Figure S1.

### Culture, identification and drug-susceptibility testing of bacterial strains

Screening cultures (throat swabs, rectal swabs and stool samples) were examined for the presence of *Pseudomonas* sp. by plating the specimens on Cetrimide agar (Becton, Dickinson and Company, France) and incubation for 48 h, at 35 °C. Bacterial identification was achieved using a linear Matrix-assisted laser desorption ionization-time of flight (MALDI-TOF) mass spectrometer (AXIMA Assurance, bioMérieux SA, France) and confirmed by MALDI-TOF mass spectrometry (Microflex LT, Bruker Daltonics, Germany). Isolates belonging to the *P. putida* group (*P. putida*, *P. monteilii*, *P. plecoglossicida*, *P. mosselii*, *P. fulva*, *P. parafulva*, *P. cremoricolorata*) and *P. aeruginosa* isolates were included in the study [30]. Strains from the *P. putida* group are referred to as “*P. putida*” in our study. In vitro antimicrobial susceptibility testing was performed with the VITEK 2 system (bioMérieux SA, France) and confirmed by Etest (bioMérieux SA, France) for meropenem. Results were interpreted according to the guidelines of the European Committee on Antimicrobial Susceptibility Testing (EUCAST), applying the clinical breakpoints for *P. aeruginosa* [31]. The first strain isolated from a patient was included in the study. *P. putida* strains from the same patient were additionally included if they differed in the antimicrobial susceptibility pattern by more than two antibiotic classes. Environmental isolates were obtained from water-associated sources (toilet, shower and basin sink) in the patients’ rooms. Sampling was performed at four different time points during the study (month 3, 8, 24, 28). Swabs were taken at a defined location and processed as described above. The molecular detection of the metallo-β-lactamase genes *bla*_IMP_ and *bla*_VIM_ was performed in all meropenem-non-susceptible isolates as described previously [32]. All strains were stored at −80 °C for further analysis.

### DNA extraction, library preparation, whole genome sequencing (WGS)

Genomic DNA was extracted from overnight bacterial cultures using the UltraClean® Microbial DNA isolation kit (MO BIO Laboratories Inc., Carlsbad, USA). Genomic DNA was sheared by Covaris M220 (Covaris, Woburn, USA) to obtain 550 bp fragments. DNA libraries were prepared with TruSeqNano DNA LT Kit (Illumina, San Diego, USA) using the standard protocol. Barcoded libraries were analyzed on the QIAxcel Advanced Instrument (Qiagen, Hilden, Germany). All libraries were sequenced at 2 × 250 bp on an Illumina MiSeq (Illumina, San Diego, USA), with the exception of two isolates (P21-B and P21_aeruginosa) in which DNA was extracted using the Qiagen Genomic-tip 100/G kit (Qiagen, Hilden, Germany) and sequencing was performed generating 2 × 300 bp paired-end reads and isolate *P. aeruginosa* P6 which was sequenced generating 2 × 50 bp on an Illumina HiSeq2000 (Illumina, San Diego, USA).

### WGS data analysis

Sequencing reads were assembled using the A5 pipeline (version 20,140,604) and SPAdes (version 3.7.0) [33, 34]. The core genomes of *P. putida* and *P. aeruginosa* were generated by Spine (version 0.1.2) using the default parameter setting except in adjusting segment length to 1000 bp resulting in a *P. putida* core genome of 2,068,252 bp and a *P. aeruginosa* core genome of 6,193,571 bp. Core genome SNPs were called using SPANDx (version 3.1) [35]. A maximum-likelihood tree was then estimated using RAxML (version 8.2.6) with a GTR substitution model and gamma distribution of rates undergoing 1000 bootstraps [36]**.** The final tree was visualized by FigTree (version 1.4.2).

The average nucleotide identity between assembled strains of the *P. putida* group was calculated using JSpecies (version 1.2.1) [37]. Whole genome sequence data of type strains from the *P. putida* group and closely related species [38] were obtained from the NCBI database: *P. putida* NBRC 14164 (NC_021505.1), *P. monteilii* NBRC 103158 = DSM 14164 (NZ_JHYV00000000.1), *P. parafulva* NBRC 16636 = DSM (GCA_000730645.1), *P. fulva* NBRC 16637 = DSM 17717 (NZ_JHYU00000000.1), *P. plecoglossicida* NBRC 103162 = DSM 15088 (NZ_JHYX00000000.1), *P. taiwanensis* DSM 21245 (NZ_AUEC00000000.1), *P. mosselii* DSM 17497 (NZ_JHYW00000000.1), *P. entomophila* str. L48 (NC_008027.1), *P. japonica* NBRC 103040 = DSM 22348 (NZ_BBIR00000000.1), *P. vranovens*is DSM 16006 (NZ_AUED00000000.1), *P. alkylphenolia* strain KL28 (NZ_CP009048.1, *P. cremoricolorata* DSM 17059 = NBRC 16634 (NZ_CP009455.1).

WGS datasets were analysed for the presence of acquired resistance genes by uploading the assembled genomes to the ResFinder 2.1 web-based analysis tool [39]. Due to errors in the genome assembly, resistance genes might be missed by this approach. Therefore, detected resistance genes were confirmed by re-mapping the quality trimmed reads against any resistance gene identified in the assemblies of the study strains using BWA-MEM [40] with a minimal mapping score of 40. The presence of plasmids was predicted from the WGS datasets, applying the web-based tool PlasmidFinder [41], Recycler [42] and plasmidSPAdes (version 3.9.0) [43]. Predicted plasmids (>2000 bp) were examined for ARGs by applying the ResFinder 2.1 web-based analysis tool. The genetic environment of the *bla*_VIM_ gene was examined in all contigs harbouring *bla*_VIM_ that originated from the WGS datasets and the predicted plasmids if *bla*_VIM_ was present. Therefore, the contigs originating from the a5 assembly were annotated using PATRIC [44], followed by a manual examination of 10 kbp upstream and downstream of the *bla*_VIM_ gene to identify neighbouring ARGs, integrons, plasmid-specific genes or transposon-related genes. Due to the limitations of the assembly in eleven *P. putida* strains and five *P. aeruginosa* strains, no *bla*_VIM_ could be identified from the FASTA files assembled with a5. In addition, the *bla*_VIM_ gene was located on contigs smaller than 10 kb in another eleven *P. putida* strains. In these cases, the FASTA generated by SPAdes was used for the analysis of the genetic environment of the *bla*_VIM_ gene and for the determination of plasmidspecific or transposon-related genes. This information and assembly quality scores are summarized in Additional file 2: Table S1 and Additional file 3: Table S2.

The genomic localization of the *bla*_VIM_ gene was determined as follows: a localisation on a plasmid was considered most likely, if one of the bioinformatic plasmid detection tools (PlasmidFinder, Recycler or plasmidSPAdes) predicted a *bla*_VIM_ harbouring contig to be a plasmid. If the respective contig was not predicted to be a plasmid and if no plasmid-specific genes were identified on the contig harbouring *bla*_VIM_, the location of the *bla*_VIM_ gene was considered chromosomal. In case the *bla*_VIM_ harbouring contig was not predicted to be a plasmid but plasmid-specific genes were observed within the nucleotide range described above, the localisation of the *bla*_VIM_ gene was considered indeterminate.

### Construction of the 16S rRNA based maximum-likelihood tree

In order to further investigate whether the core genome clustering of the *P. putida* group strains was based on their affiliation to the different species of this group, we extracted the 16S rRNA sequences from each assembly and generated an clustalW alignment [45] after integration of reference sequences (*P. putida* AB008001, *P. fulva* AB060131, *P. parafulva* AB060133, *P. plecoglossicida* AB 009457, *P. mosselii* AF072688, *P. alkylphenolica* AY324319, *P. vranovensis* AY970951, *P. cremoricolorata* AB060137, *P. japonica* AB126621, *P. entomophila* AB541974, *P. taiwanensis* EU857417, *P. monteilii* AB211409) from the SILVA database (https://www.arb-silva.de/). The 16S rRNA maximum-likelihood tree was generated using RAxML (version 8.2.6) with a GTR substitution model and gamma distribution of rates undergoing 1000 bootstraps [36].

### Epidemiological data

Epidemiological data were obtained to detect potential transmissions between patients that were colonized with strains from the same genetic clusters or to localize a potential environmental source. A Time-Place-Sequence algorithm characterized transmission likelihood as described previously with minor modifications [29]. The probability of transmission from patient 1 to patient 2 was based on three criteria. Criterion 1 was considered fulfilled if the patients were hospitalized on the same ward for at least 24 h (possible transmission); criterion 2 was considered fulfilled if patient 2 stayed in the same room up to 3 months (probable transmission) or more than 3 months (possible transmission) after the first patient. Criterion 3 was fulfilled when both patients shared the same room at the same time with an overlap of at least 24 h (probable transmission). For identification of a potential environmental source, the rooms, wards and transfers during the hospital stay were documented for patients colonized with a *bla*_VIM_ positive *P. putida* strain.

## Results

Rectal swabs, throat swabs and stool samples were obtained from a weekly active screening culture program to detect *Pseudomonas sp*. colonization. A total of 21,373 specimens including 10,528 rectal swabs, 8904 throat swabs and 1941 stool samples from 2276 patients were analysed over a period of 28 months. In total, 89 *P. putida* strains were isolated from 85 patients and were further characterized. These strains originated from rectal swabs (*n* = 46), throat swabs (*n* = 26), and stool samples (*n* = 17). The resistance characteristics of the *P. putida* strains investigated are summarized in Table 1. Highest resistance rates were observed for piperacillin (68.5%), piperacillin-tazobactam (66.3%), followed by meropenem (64.1%), ceftazidime (57.3%), ciprofloxacin (47.2%), cefepime (43.8%) and gentamicin (14.6%). A *bla*_VIM_ gene was detected in 41 of the 62 meropenem-non-susceptible strains (66.1%). These 41 strains were isolated from 40 patients. The majority of *bla*_VIM_ positive isolates were resistant to cefepime (92.2%) in contrast to *bla*_VIM_ negative isolates with a susceptibility rate of 97.9%. The *bla*_VIM_ positive isolates were generally resistant to most antibiotics, with the exception of gentamicin, to which 28 of 41 isolates (68.3%) were susceptible, suggesting the presence of at least two genetically different *bla*_VIM_ positive *P. putida* strains.

**Table 1:** Susceptibility profiles of 89 clinical isolates belonging to the *P. putida* group. Strains were isolated from throat swabs (*n* = 26), rectal swabs (*n* = 46) and stool (*n* = 17) active screening cultures from 85 patients over period of 28 months

Antimicrobial agent	*bla*_vim_ negative (*n* = 48) n(%)			*bla*_vim_ positive (*n* = 41) n(%)			Totla (*n* = 89) n (%)		
	R	I	S	R	I	S	R	I	S
PIP	20 (41.7)	–	28 (58.3)	41 (100)	–		61 (68.5)	–	28 (31.5)
TZP	18 (37.5)	–	30 (62.5)	41 (100)	–		59 (66.3)	–	30 (33.7)
CAZ	10 (20.8)	–	38 (79.2)	41 (100)	–		51 (57.3)	–	38 (42.7)
FEP	1 (2.1)	–	47 (97.9)	38 (92.2)	–	3 (7.8)	39 (43.8)	–	50 (56.2)
MEM	16 (33.3)	5 (10.4)	27 (56.3)	41 (100)			57 (64.1)	5 (5.6)	27 (30.3)
CIP	2 (4.2)	3 (6.3)	43 (89.5)	40 (97.6)	1 (2.4)		42 (47.2)	4 (4.5)	43 (48.3)
GEN	0 (0)	–	48 (100)	13 (31.7)	–	28 (68.3)	13 (14.6)	–	76 (85.4)

*PIP* piperacillin, *TZP* piperacillin-tazobactam, *CAZ* ceftazidime, *FEP* cefepime, *MEM* meropenem, *CIP* ciprofloxacin, *GEN* gentamicin, *S* susceptible, *I* intermediate, *R* resistant

### Genetic relatedness of *bla*_VIM_ positive *P. putida* strains

**Fig. 1:**
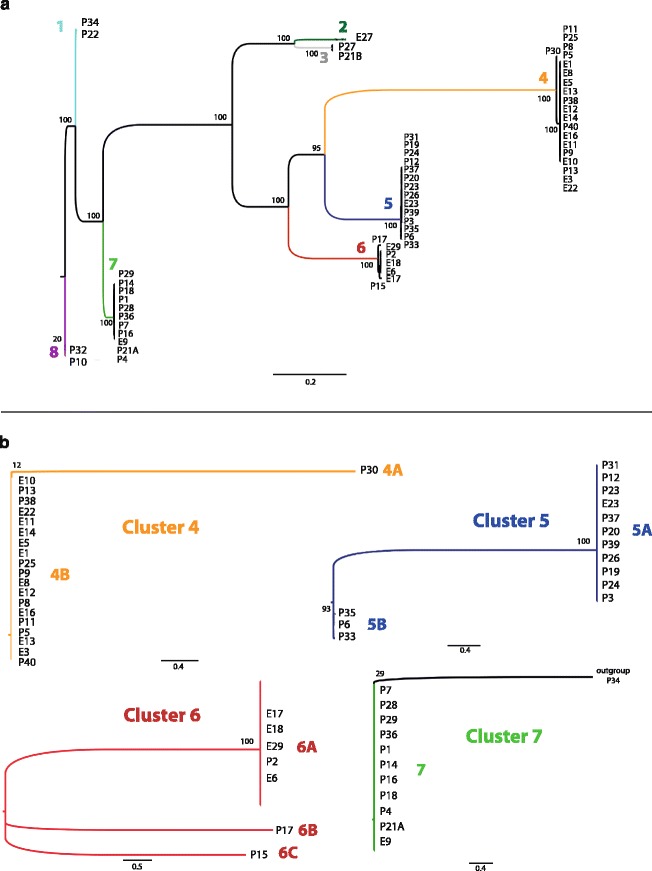
**a** Core genome maximum-likelihood phylogeny of *bla*_VIM_ positive *P. putida* isolates comprising 41 *bla*_VIM_ positive patient isolates (“P” as first letter) and 18 *bla*_VIM_ positive environmental isolates (“E” as first letter). The strains could be divided into 8 different phylogenetic clusters. The numbers displayed at the nodes are bootstrap values. The scale bar represents the expected number of changes per site. **b** For each of the clusters 4-7, a separate core genome maximum-likelihood tree was constructed and allowed further resolution into subclusters. An outgroup strain (P34 from cluster 1) was introduced into cluster 7 to ensure a proper visualisation of the high similarity between the cluster members

In order to characterize genetic relatedness, whole genome sequencing of the 41 *bla*_VIM_ positive patient isolates and 18 *bla*_VIM_ positive environmental isolates was performed. The maximum-likelihood tree showed a separation into 8 distinct clusters (Fig. 1a). Cluster 4 comprised most strains (*n* = 20), followed by cluster 5 (*n* = 14), cluster 7 (*n* = 11) and cluster 6 (*n* = 7). The other four clusters contained only one or two isolates. In order to increase the resolution of the closely related strains in clusters 4, 5, 6, and 7, a separate maximum likelihood phylogeny was constructed with those strains. Thereby, clusters 4, 5 and 6 could be further divided into subclusters (Fig. 1b). Interestingly, clusters 5 and 7 predominantly contained isolates originating from patients, whereas clusters 4 and 6 comprised both isolates from patients and the environment. In order to assess whether the different phylogenetic clusters represent the different species of the *P. putida* group, the average nucleotide identity of the study isolates and closely related type strains was performed (Additional file 4: Table S3 and Additional file 5: Table S4). With the exception of cluster 1, 7, 8 which were related to *P. monteilii,* none of the clusters showed ANI values >95% with any of the reference type strain genomes (Additional file 5: Table S4). Additionally, the WGS based maximum-likelihood phylogeny was compared to a 16S rRNA based maximum-likelihood phylogeny tree including 16S rRNA reference strain sequences (Additional file 6: Figure S2). The strains of the WGS clusters 2, 3, 4, 5 and 6 arranged in an equal manner. While the clusters 1, 8 and three strains of cluster 7 could not be further resolved based on the 16S RNA maximum-likelihood phylogeny, they closely clustered with the *P. monteilii* reference, thus confirming the observations of the ANI analysis.

### Timeline of colonization and transmission routes

**Fig. 2:**
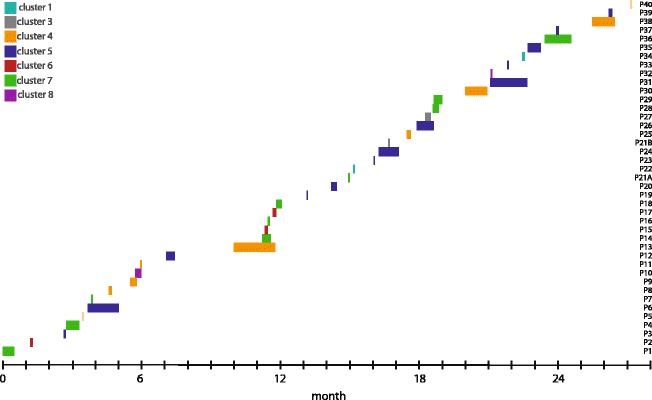
Timeline of *bla*_VIM_ positive *P. putida* colonization in 40 patients during the study period. The different phylogenetic clusters for patients P1-P40 are displayed. The major clusters were continuously observed during the study period, without accumulation of a certain cluster at a given point in time. In P21, two *P. putida* strains and one *P. aeruginosa* strain were isolated (P21A, cluster 7 and P21B cluster 3 and *P. aeruginosa* P21 Additional file 8: Figure S3)

*Bla*_VIM_ positive *P. putida* strains from different phylogenetic clusters were encountered continuously in the 40 colonized patients over the 28 months as displayed in Fig. 2. There was no accumulation of a certain genetic cluster observed at a given point in time.

In order to establish transmission routes, epidemiological data were linked to patients and environmental samples within the same genetic cluster. This enabled us to detect 12 cases of probable transmissions (summarized in Additional file 7: Table S5). According to our analysis, plausible routes of transmission include the following: i) Patient colonization following exposure to an environmental source was considered probable if environmental isolates that belonged to the same phylogenetic cluster as the isolate of the patient were recovered before the patient stayed in the room (e.g. cluster 4B, P5). ii) Patient-to-patient transmission was considered probable when the patients stayed in the same room at the same time (e.g. cluster 7 P28/29) and when both patients were sequentially colonized with *P. putida* isolates from the same phylogenetic cluster. For patients that stayed on the same ward at the same time or consecutively in the same room, a transmission from an environmental source was considered possible. In all cases, transmissions via hospital personnel could be another route and cannot be excluded.

### Genetic relatedness of *P. aeruginosa bla*_VIM_ positive strains isolated from the study cohort and environment sources

During the study period, *bla*_VIM_ positive *P. aeruginosa* strains were isolated from seven patients of the study cohort. The strains were further characterized in order to determine their resistance gene content and to evaluate whether genetic resistance determinants were shared between *P. putida* and *P. aeruginosa* strains isolated from the same patient cohort and environmental sources. All seven *bla*_VIM_ positive *P. aeruginosa* isolates were resistant to gentamicin, piperacillin, piperacillin-tazobactam, ceftazidime, cefepime, meropenem and ciprofloxacin. All strains remained susceptible to colistin.

In addition, 10 *bla*_VIM_ positive *P. aeruginosa* environmental isolates were included in the study. In contrast to the genetic diversity observed in *P. putida* isolates, all patient and environmental *bla*_VIM_ positive *P. aeruginosa* strains were genetically highly similar as illustrated in the maximum-likelihood phylogenetic tree in Additional file 8: Figure S3.

### Characterization of antibiotic resistance genes in *bla*_VIM_ positive *P. putida* and *P. aeruginosa* isolates

**Fig. 3:**
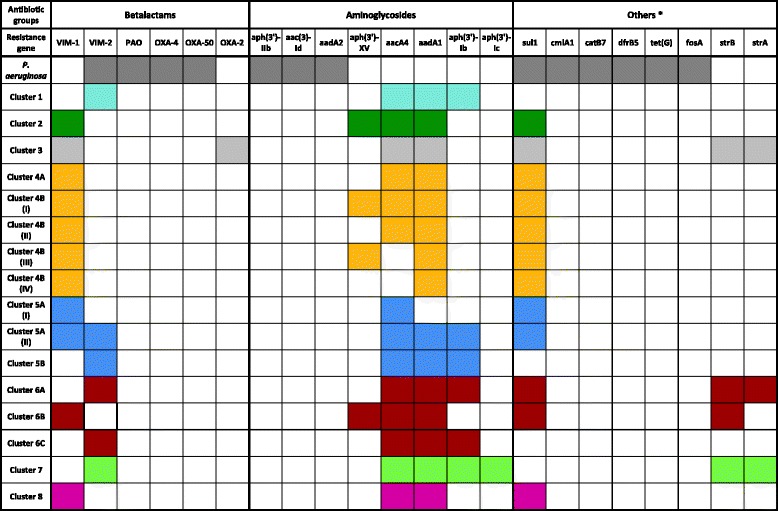
Overview of ARG content in the phylogenetic clusters of *bla*_VIM_ positive *P. putida* and *P. aeruginosa* strains. Strains within a cluster usually contained the same ARGs, with the exception of clusters 4B and 5A as indicated by the shaded fields. In cluster 4B both genes, *aph*(3′)-XV and *aacA4* were present in P5, P8, P11, E1, E3, E5, E11, E13 (Cluster 4B I). *Aph*(3′)-XV was missing in the isolates P13, P38, P40, E10, E12, E14 (cluster 4B II), and *aacA4* was missing in the isolates P9, P25, P40, E16 and E22 (cluster 4B III). In P40 both genes were absent (cluster 4B IV). In cluster 5A, two different types of ARG patterns were observed. Here, the strains P3, P19, P24, P26, P39 (cluster 5A II) contained a *bla*_VIM-2_, *aadA1* and *aph3-Ib* gene in addition to the other ARGs of that cluster

The presence of ARGs was explored in all isolates in order to assess ARG transfer between *P. putida* and *P. aeruginosa bla*_VIM_ positive isolates. The presence and absence of ARGs is shown in Fig. 3. All *P. putida* strains from a phylogenetic cluster harboured the same ARGs with the exceptions of cluster 4B and cluster 5A, where different ARG patterns were observed.

The *bla*_VIM-1_ gene was present in 7 phylogenetic clusters and subclusters (2, 3, 4A, 4B, 5A, 6B and 8) while the *bla*_VIM-2_ gene was detected in 6 phylogenetic clusters and subclusters (1, 5A, 5B, 6A, 6C and 7). Of note, five strains in cluster 5A contained both, *bla*_VIM-1_ and *bla*_VIM-2_ genes (P3, P19, P24, P26, P39). In the VIM-1 groups, the *bla*_VIM-1_ gene was often co-localized with aminoglycosides resistance genes and was either predicted to be on a plasmid (clusters 2, 4A, 4B, 6B and 8) or on the chromosome (clusters 3 and 5A) (Additional file 9: Table S6). The *bla*_VIM-2_ gene was also often co-localized with aminoglycoside ARGs, but was only predicted to be located on a plasmid in one strain (P4) of cluster 7 (Additional file 9: Table S6).

In contrast to the diverse ARG situation in the *P. putida* isolates, all 17 *P. aeruginosa* isolates harboured the beta-lactamases *bla*_VIM-2_, *bla*_PAO_, *bla*_OXA-4_, *bla*_OXA-50_ in addition to aminoglycoside resistance enzymes (*aph(3′)-IIb*, *aac(3)-Id* and *aadA2*) and other ARG groups (*sul1*, *cmlA1*, *catB7*, *dfr5*, *tet(G)* and *fosA*). The *bla*_VIM-2_ was either directly flanked by the ARGs *dfrB5* and *aac(3)-Id* or was located alone with no adjacent ARGs in the direct genetic environment. No plasmid-specific genes were detected 10 kb upstream and downstream from the *bla*_VIM-2_ location on the respective contig. Furthermore, no *bla*_VIM-2_ harbouring plasmids were predicted by PlasmidFinder, Recycler and plasmidSPAdes (Additional file 9: Table S6), which provides evidence for a chromosomal location of *bla*_VIM-2_ in those strains. Furthermore, despite sharing the *bla*_VIM-2_ and *sul* gene, no other resistance genes were shared between *P. aeruginosa* and *P. putida* strains. Both findings clearly reflect the absence of epidemiological evidence for the exchange of genetic elements harbouring ARG between *P. aeruginosa* and *P. putida*.

Interestingly, study patient 21 was colonized with a *P. putida* isolate (P21A, cluster 7) prior to the simultaneous isolation of a *P. putida* strain (P21B, cluster 3) and *P. aeruginosa* strain P21 from the same stool specimen. Apart from *bla*_VIM-2_ (P21A) and *sul* (P21B), both *P. putida* strains shared no further common ARGs with the *P. aeruginosa* strain. Thus, our initial suspicions, based only on epidemiological data, that an exchange of comprehensive genetic mobile elements and horizontal spread of *bla*_VIM_ had occurred, were clearly disproven.

## Discussion

Infections and colonization with *P. putida* have been reported from various sources [2–9]; however, to the best of our knowledge, the study presented here is the first to systematically evaluate the frequency of *P. putida* colonization in a haemato-oncological patient population combined with a comprehensive genetic characterization of the isolates. During the 28-month study period, active screening cultures that included 2276 patients were performed and resulted in the isolation of 89 *P. putida* strains from 85 patients. Among these isolates, the rate of *bla*_VIM_ positive *P. putida* was very high (46.1%). This is particularly worrisome as *bla*_VIM_ positive low pathogenic bacteria may transfer the *bla*_VIM_ gene to more pathogenic bacteria in a colonized patient, thereby increasing the risk to an individual patient of becoming infected with a modified strain that is both resistant and highly pathogenic. These modified strains could then be spread in the hospital environment and could build up a reservoir that constitutes a continuous exposure to patients. Moreover, such strains could become the source strains for a further *bla*_VIM_ gene transfer to other bacterial species in patient-related hospital environments, potentially causing a chain reaction that may be impossible to control once in progress.

Several studies have provided evidence for the transfer of ARGs from *P. putida* to *P. aeruginosa* and have described the role of *P. putida* as a reservoir and exchange platform for ARGs [12, 18, 28]. If these findings were proven true, we would then need to target *P. putida* as well as the more pathogenic *P. aeruginosa* in a high-risk patient setting by using appropriate infection control measures. Lee et al. examined 43 *bla*_VIM-2_ positive *P. aeruginosa* and 9 *bla*_VIM-2_ positive *P. putida* clinical isolates. The flanking regions of one *P. aeruginosa* isolate and one *P. putida* isolate were sequenced revealing two different integron structures. Applying the filter mapping method, carbapenem resistance could be successfully transferred from 6 of the *P. aeruginosa* isolates and 2 of the *P. putida* isolates to a *P. aeruginosa* recipient. However, no plasmid harbouring the *bla*_VIM-2_ gene could be detected in the recipient strain [18]. Juan et al. examined 8 *P. putida* and 11 *P. aeruginosa* clinical isolates from one hospital [28]. Both *P. putida* and *P. aeruginosa* isolates possessed an identical transposon that contained a *bla*_VIM-2_ integron, thus providing evidence for a horizontal gene transfer between the two species. In addition, plasmids harbouring *bla*_VIM-2_ from three *P. putida* clones were successfully transferred to the laboratory *P. aeruginosa* strain PAO1. Nevertheless, the *bla*_VIM-2_ genes in the clinical *P. aeruginosa* strains had a chromosomal location in all but one clone. Juan et al. suggested the concurrent presence of the *bla*_VIM-2_ gene on the chromosome in that particular *P. aeruginosa* strain. But it must be noted that the plasmid harbouring *bla*_VIM-2_ differed in its genetic pattern from the plasmid harbouring *bla*_VIM-2_ of the *P. putida* isolates [28]. Therefore, the data present epidemiological evidence for the horizontal gene transfer of a transposon structure containing *bla*_VIM-2_, but no evidence for the transfer of a complete identical plasmid between the species [28]. In another study, also conducted on the Canary Islands, six MBL-producing strains were examined, including two *bla*_IMP-15_ positive *P. aeruginosa* and two *bla*_IMP-15_ positive *P. putida* isolates. The simultaneous merging of the two species harbouring the rarely encountered *bla*_IMP-15_ gene suggested a horizontal gene transfer, but the location of the genes was chromosomal and plasmid transfer experiments did not result in resistant recipients [12].

In the study presented here, *P. putida* strains from our hospital formed a heterogeneous group comprising eight phylogenetic clusters. Interestingly, the main clusters were distributed over the whole study period (Fig. 2), without an accumulation of a certain cluster at a given point in time. Transmission analysis revealed that environment-to-patient transmission as well as patient-to-patient transmission was likely to have occurred, which is quite similar to the transmission routes observed during a *P. aeruginosa* outbreak in our hospital [29].

A total of 11 different ARGs were detected in our *P. putida* strains (Fig. 3). Strains of the same phylogenetic cluster usually contained the same ARGs, indicating a clonal spread rather than horizontal gene transfer of ARGs. Exceptions to this were found in clusters 4B and 5A where strains differed in their ARG content. This suggests that strains in these clusters acquired resistance genes via gene transfer at some point. Of particular note is what we found in cluster 5A, in which five isolates harboured both a *bla*_VIM-1_ and a *bla*_VIM-2_ gene (Fig. 3, Cluster 5A II). However, there was no evidence for the presence of ARGs on plasmids in the strains of this cluster. But plasmids were identified in strains belonging to other clusters. Plasmids carrying *bla*_VIM-1_ were predicted in 24 *P. putida* from five phylogenetic clusters and a plasmid carrying *bla*_VIM-2_ in one strain from cluster 7. Since the *bla*_VIM-2_ gene was also detected in our *P. aeruginosa* strains, we hypothesized that an ARG transfer might have occurred between both species. But despite the detection of the plasmid harbouring *bla*_VIM-2_ in one *P. putida*, we could not detect any further shared ARGs between both species besides the *bla*_VIM-2_ and *sul* genes. No plasmids harbouring *bla*_VIM-2_ were predicted in *P. aeruginosa* isolates.

To date, there are both limitations and challenges in applying WGS to analyse ARGs and predict plasmids. First, the quality of assembly is a crucial factor for a general analysis of ARGs and for the analysis of the genomic environment. ARGs that are incomplete or incorrectly assembled might not be found in applying tools based on searches against ARG databases (e.g. ResFinder). In order to minimize this limitation, two different assemblers were applied and both assemblies searched for the presence of ARGs. The presence of identified ARGs was subsequently confirmed by remapping the unassembled reads against these ARGs. Another limitation is that the location of a certain ARG (i.e. on a chromosome or plasmid) can only be predicted. The ability to identify a plasmid depends on several factors like genome coverage, read length and presence of repetitive sequences on the plasmid [46]. Due to these limitations, we applied three different bioinformatics tools (PlasmidFinder, plasmidSPAdes and recycler) to our dataset in order to augment plasmid detection [41–43]. Furthermore, plasmids predicted using these tools were only interpreted in combination with a manual search for annotated plasmid-specific genes in the genetic environment of contigs that harboured the *bla*_VIM_ gene. Additional laboratory experiments, e.g. plasmid preparations and transconjugation experiments, might potentially provide further insights. However, these experimental settings are hampered by the fact, that bacterial isolates can contain multiple plasmids of various sizes [47], making it nearly impossible to interpret the data in terms of ARG localisation, especially in studies including many distinct isolates. Moreover, even in the case of an observed transfer in transconjugation experiments, it can be tricky to infer the direction of an ARG transmission as it has occurred beyond laboratory standardization in a clinical setting. Considering these limitations, we are still confident that the application of our multistep-analysis approach based on WGS data did produce a comprehensive overview of the ARGs and predicted plasmids in our study isolates.

## Conclusions

Based on the findings presented above, there is no evidence for a horizontal gene transfer of ARGs on transposons or plasmids from members of the *P. putida* group to *P. aeruginosa* in our hospital. Although ARG transfer might have occurred below our detection limit, the transfer of ARGs into a *P. aeruginosa* strain that is successfully adapted to surviving in the hospital environment and that is capable of colonizing patients seems to be a rare event and was not observed during the study period of 28 months including a total of 21,373 active screening cultures. Nevertheless, further studies need to be conducted to understand the mechanisms and to monitor the occurrence of horizontal gene transfer, not only between species of the same genus but also between more distinct bacterial taxa, particularly between *Pseudomonas* sp. and the clinically relevant *Enterobacteriaceae*. While we consider the latter scenario as indeed possible and a potential event with a severe impact, we can also state at this point that the isolation of *bla*_VIM_ positive *Enterobactericeae* is still very uncommon in our hospital, which is the reason why we did not address this issue in our study. In summary, based on the evidence provided here, we do not see the necessity of applying particular infection control measures for patients colonized with *bla*_VIM_ positive *P. putida* strains in our hospital.

## Additional files

Additional file 1: Figure S1. Workflow of the study design and strain inclusion criteria. (PDF 151 kb)

Additional file 2: Table S1. Overview of *P. putida* assembly statistics and genome coverage based on the assembly obtained for the a5 assembler. (DOCX 17 kb)

Additional file 3: Table S2. Overview of *P. aeruginosa* assembly statistics and genome coverage based on the assembly obtained for the a5 assembler. (DOCX 14 kb)

Additional file 4: Table S3. Average of the nucleotide identity of the *P. putida* study strains and closely related species. (XLSX 35 kb)

Additional file 5: Table S4. Overview of the closest related type strains based on the average nucleotide identity (ANI). (DOCX 16 kb)

Additional file 6: Figure S2. 16S rRNA based maximum-likelihood tree of the *P. putida* group isolates and reference strains. The numbers displayed at the nodes are bootstrap values. The scale bar represents the expected number of changes per site. (PDF 456 kb)

Additional file 7: Table S5. Summary of epidemiological data of the *P. putida* isolates from the different NGS clusters. (DOCX 18 kb)

Additional file 8: Figure S3. Core genome maximum-likelihood phylogeny of 17 blaVIM-2 positive *P. aeruginosa* strains isolated from patients (*n* = 7, “P” as first letter) and water-related environmental sources (*n* = 10, “E” as first letter). All isolates are genetically highly similar. An outgroup strain (blaIMP-8 positive *P. aeruginosa* [29]) was introduced to ensure a proper visualisation of the strains’ sequence similarity and relatedness. The scale bar represents the expected number of changes per site. *P21 was also colonized with two different *P. putida* strains P21A and P21B. (PDF 295 kb)

Additional file 9: Table S6. Overview of predicted plasmid harbouring a *bla*_VIM_ gene and the characteristics of the genetic *bla*_VIM_ environment. (DOCX 31 kb)

## Abbreviations

ARG: Antibiotic resistance genes
ASC: Active screening cultures
*Bla*: β-lactamase
CAZ: Ceftazidime
CIP: Ciprofloxacin
EUCAST: European committee on antimicrobial susceptibility testing
FEP: Cefepime
GEN: Gentamicin
I: Intermediate
ICU: Intensive care units
IMP: Imipenemase
MALDI-TOF: Matrix-assisted laser desorption ionization-time of flight
MBL: Metallo-β-lactamase
MEM: Meropenem
NDM: New Delhi metallo-β-lactamase
P.: Pseudomonas
PIP: Piperacillin
R: Resistant
S: Susceptible
TZP: Piperacillin-tazobactam
VIM: Verona integron-encoded metallo-β-lactamase
WGS: Whole genome sequencing
