# Supplementary material for: Genomic characterisation of clinical and environmental Pseudomonas putida group strains and determination of their role in the transfer of antimicrobial resistance genes to Pseudomonas aeruginosa
Source: BMC Genomics. 2017 Nov 10;18:859. doi: 10.1186/s12864-017-4216-2 (PMC5681832; doi:10.1186/s12864-017-4216-2)
Supplement: Supplementary file 1 — Workflow of the study design and strain inclusion criteria. (PDF 151 kb) [file 12864_2017_4216_MOESM1_ESM.pdf]

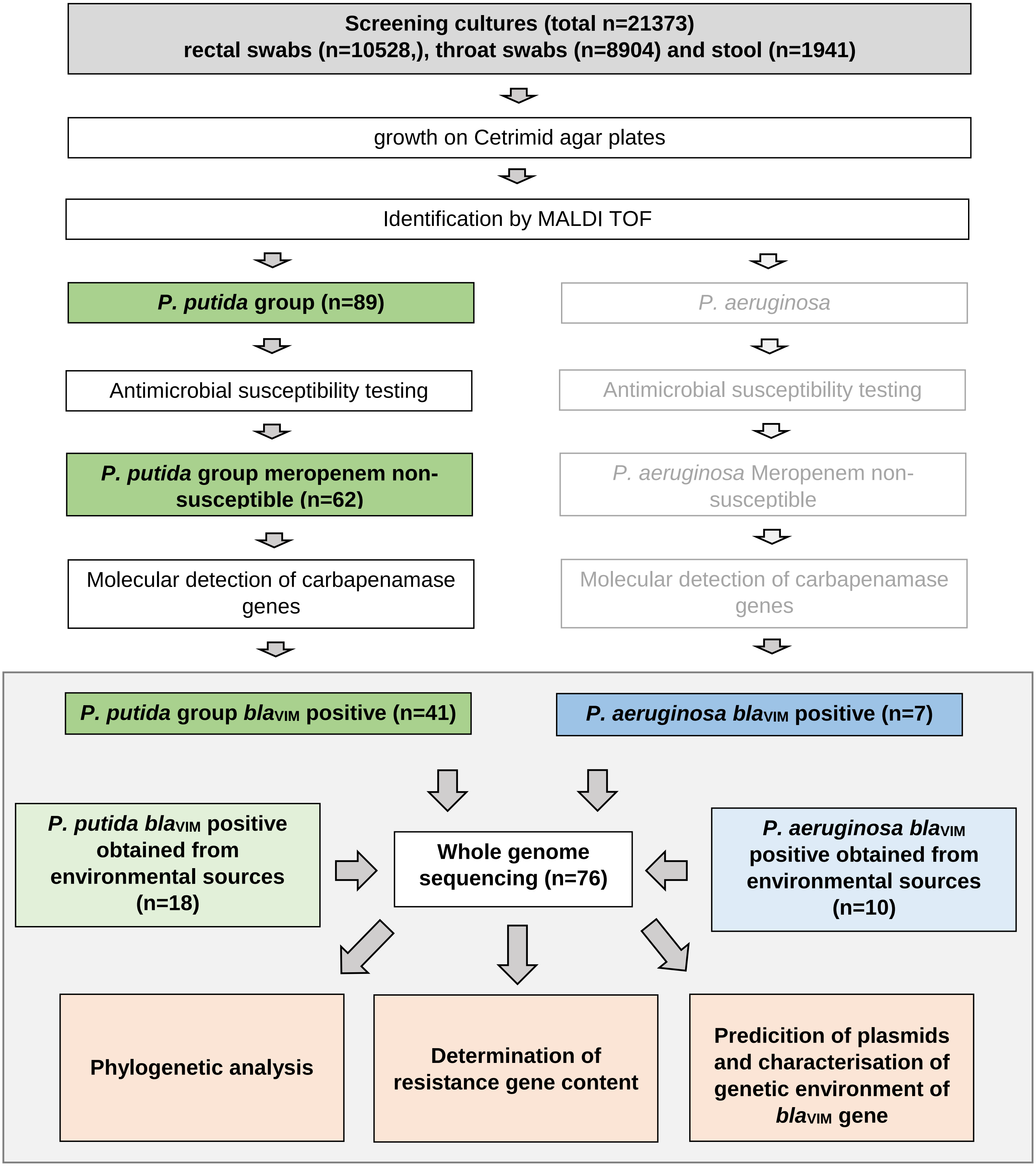
Screening cultures (total n=21373)
rectal swabs (n=10528,), throat swabs (n=8904) and stool (n=1941)
growth on Cetrimid agar plates
Identification by MALDI TOF
P. putida group (n=89)
P. aeruginosa
Antimicrobial susceptibility testing
Antimicrobial susceptibility testing
P. putida group meropenem non-susceptible (n=62)
P. aeruginosa Meropenem non-susceptible
Molecular detection of carbapenamase genes
Molecular detection of carbapenamase genes
P. putida group blaVIM positive (n=41)
P. aeruginosa blaVIM positive (n=7)
P. putida blaVIM positive obtained from environmental sources (n=18)
Whole genome sequencing (n=76)
P. aeruginosa blaVIM positive obtained from environmental sources (n=10)
Phylogenetic analysis
Determination of resistance gene content
Predicition of plasmids and characterisation of genetic environment of blaVIM gene
