# Supplementary material for: Genomic characterisation of clinical and environmental Pseudomonas putida group strains and determination of their role in the transfer of antimicrobial resistance genes to Pseudomonas aeruginosa
Source: BMC Genomics. 2017 Nov 10;18:859. doi: 10.1186/s12864-017-4216-2 (PMC5681832; doi:10.1186/s12864-017-4216-2)
Supplement: Supplementary file 2 — Overview of P. putida assembly statistics and genome coverage based on the assembly obtained for the a5 assembler. (DOCX 17 kb) [file 12864_2017_4216_MOESM2_ESM.docx]

| **ID** | **no. of contigs** | **N50** | **%GC** | **concatenated genome bp** | **x coverage** | **comment** |
| --- | --- | --- | --- | --- | --- | --- |
| P1 | 56 | 261785 | 62,14 | 6243024 | 23 |  |
| P2 | 87 | 192156 | 61,65 | 6845077 | 32 | SPAdes assembly (genetic environment bla_VIM_) |
| P3 | 42 | 432127 | 61,96 | 6452818 | 29 | SPAdes assembly (genetic environment bla_VIM_) |
| P4 | 47 | 299200 | 62,16 | 6250816 | 24 |  |
| P5 | 93 | 248370 | 61,38 | 6671965 | 36 |  |
| P6 | 52 | 315558 | 61,91 | 6471241 | 24 |  |
| P7 | 59 | 256413 | 62,10 | 6331660 | 36 |  |
| P8 | 82 | 242743 | 61,40 | 6691602 | 30 |  |
| P9 | 93 | 207662 | 61,40 | 6699557 | 27 |  |
| P10 | 61 | 219554 | 61,72 | 6019197 | 22 | SPAdes assembly (genetic environment bla_VIM_) |
| P11 | 89 | 223690 | 61,38 | 6712377 | 27 |  |
| P12 | 34 | 510112 | 62,00 | 6385092 | 23 |  |
| P13 | 88 | 242742 | 61,38 | 6716182 | 27 |  |
| P14 | 53 | 249504 | 62,10 | 6330801 | 31 |  |
| P15 | 53 | 248745 | 61,72 | 6796620 | 27 |  |
| P16 | 49 | 233146 | 62,10 | 6331750 | 26 |  |
| P17 | 67 | 239796 | 61,71 | 6625500 | 36 |  |
| P18 | 292 | 40270 | 61,16 | 7384853 | 23 |  |
| P19 | 33 | 509454 | 61,97 | 6716182 | 65 | SPAdes assembly (genetic environment bla_VIM_) |
| P20 | 27 | 802951 | 62,02 | 6349284 | 51 |  |
| P21A | 40 | 330659 | 62,19 | 6189136 | 17 | SPAdes assembly (genetic environment bla_VIM_) |
| P21B | 60 | 195755 | 63,75 | 6312678 | 141 | SPAdes assembly (genetic environment bla_VIM_) |
| P22 | 76 | 265650 | 61,48 | 6364009 | 35 |  |
| P23 | 31 | 437623 | 61,99 | 6234951 | 48 | SPAdes assembly (genetic environment bla_VIM_) |
| P24 | 36 | 527316 | 61,97 | 6450892 | 50 | SPAdes assembly (genetic environment bla_VIM_) |
| P25 | 79 | 238779 | 61,40 | 6651626 | 26 |  |
| P26 | 31 | 796538 | 61,97 | 6450658 | 54 | SPAdes assembly (genetic environment bla_VIM_) |
| P27 | 52 | 198362 | 63,76 | 6308351 | 50 | SPAdes assembly (genetic environment bla_VIM_) |
| P28 | 43 | 347843 | 62,10 | 6393980 | 46 |  |
| P29 | 50 | 277066 | 62,10 | 6389252 | 46 |  |
| P30 | 118 | 108881 | 61,31 | 6512857 | 18 | SPAdes assembly (genetic environment bla_VIM_) |
| P31 | 28 | 431936 | 62,00 | 6386415 | 50 |  |
| P32 | 42 | 271507 | 61,94 | 5804796 | 31 |  |
| P33 | 52 | 400620 | 61,94 | 6588430 | 36 |  |
| P34 | 95 | 193659 | 61,49 | 6449513 | 13 |  |
| P35 | 92 | 174419 | 61,91 | 6452771 | 11 |  |
| P36 | 43 | 335349 | 62,14 | 6237330 | 39 |  |
| P37 | 24 | 1546087 | 62,00 | 6386280 | 44 |  |
| P38 | 82 | 248358 | 61,38 | 6716824 | 48 |  |
| P39 | 27 | 513055 | 61,97 | 6451547 | 96 | SPAdes assembly (genetic environment bla_VIM_) |
| P40 | 101 | 192679 | 61,38 | 6666272 | 16 |  |
| E1 | 85 | 261140 | 61,40 | 6701191 | 77 | SPAdes assembly (genetic environment bla_VIM_) |
| E3 | 95 | 256117 | 61,39 | 6673252 | 65 |  |
| E5 | 93 | 261144 | 61,39 | 6703944 | 67 |  |
| E6 | 92 | 163586 | 61,65 | 6848160 | 65 | SPAdes assembly (genetic environment bla_VIM_) |
| E8 | 98 | 260451 | 61,39 | 6710437 | 60 |  |
| E9 | 40 | 305590 | 62,16 | 6251699 | 72 |  |
| E10 | 102 | 256066 | 61,39 | 6712021 | 88 |  |
| E11 | 88 | 253619 | 61,38 | 6754996 | 71 |  |
| E12 | 90 | 238779 | 61,39 | 6708448 | 56 |  |
| E13 | 97 | 253289 | 61,38 | 6676280 | 73 |  |
| E14 | 111 | 201908 | 61,38 | 6721289 | 66 |  |
| E16 | 144 | 130192 | 61,38 | 6699838 | 21 | SPAdes assembly (genetic environment bla_VIM_) |
| E17 | 83 | 196800 | 61,65 | 6851013 | 42 | SPAdes assembly (genetic environment bla_VIM_) |
| E18 | 83 | 197861 | 61,64 | 6851324 | 46 |  |
| E22 | 76 | 267479 | 61,39 | 6756760 | 119 |  |
| E23 | 27 | 527184 | 62,02 | 6351610 | 71 |  |
| E27 | 95 | 198506 | 63,09 | 6961254 | 91 |  |
| E29 | 79 | 237412 | 61,65 | 6851083 | 63 | SPAdes assembly (genetic environment bla_VIM_) |

**Table S1. Overview of *P. putida* assembly statistics and genome coverage based on the assembly obtained for the a5 assembler.** In the comment it is indicated, for which isolates the SPAdes assembly was used to examine the genetic environment of the *bla*_VIM_ gene.
