# Supplementary material for: Genomic characterisation of clinical and environmental Pseudomonas putida group strains and determination of their role in the transfer of antimicrobial resistance genes to Pseudomonas aeruginosa
Source: BMC Genomics. 2017 Nov 10;18:859. doi: 10.1186/s12864-017-4216-2 (PMC5681832; doi:10.1186/s12864-017-4216-2)
Supplement: Supplementary file 3 — Overview of P. aeruginosa assembly statistics and genome coverage based on the assembly obtained for the a5 assembler. (DOCX 14 kb) [file 12864_2017_4216_MOESM3_ESM.docx]

| **ID** | **no. of contigs** | **N50** | **% GC** | **concatenated genome bp** | **coverage** | **comment** |
| --- | --- | --- | --- | --- | --- | --- |
| P1_aeruginosa | 293 | 146738 | 65,72 | 7138886 | 170 | Hiseq sequence, SPAdes assembly |
| P2_aeruginosa | 58 | 295457 | 65,68 | 7193962 | 27 |  |
| P3_aeruginosa | 58 | 373610 | 65,68 | 7196766 | 20 |  |
| P4_aeruginosa | 60 | 303776 | 65,67 | 7201951 | 27 |  |
| P5_aeruginosa | 64 | 354084 | 65,67 | 7200789 | 45 |  |
| P6_aeruginosa | 107 | 354334 | 65,53 | 7231953 | 107 |  |
| P21 | 55 | 373710 | 65,68 | 7202020 | 157 |  |
| E2 | 68 | 373610 | 65,68 | 7193410 | 57 | SPAdes assembly (genetic environment *bla*_VIM_) |
| E4 | 64 | 309425 | 65,68 | 7195775 | 66 | SPAdes assembly (genetic environment *bla*_VIM_) |
| E7 | 72 | 346247 | 65,68 | 7196744 | 64 |  |
| E15 | 67 | 324669 | 65,68 | 7199236 | 57 |  |
| E19 | 61 | 325966 | 65,68 | 7200699 | 32 | SPAdes assembly (genetic environment *bla*_VIM_) |
| E20 | 65 | 309236 | 65,68 | 7194505 | 53 |  |
| E24 | 61 | 344094 | 65,68 | 7195701 | 53 | SPAdes assembly (genetic environment *bla*_VIM_) |
| E25 | 63 | 274289 | 65,68 | 7196954 | 41 |  |
| E26 | 66 | 294936 | 65,68 | 7199554 | 51 |  |
| E28 | 59 | 295484 | 65,69 | 7189882 | 53 |  |

**Table S2. Overview of *P. aeruginosa* assembly statistics and genome coverage based on the assembly obtained for the a5 assembler.** In the comment it is indicated, for which isolates the SPAdes assembly was used to examine the genetic environment of the *bla*_VIM_ gene.
