# Supplementary material for: Genomic characterisation of clinical and environmental Pseudomonas putida group strains and determination of their role in the transfer of antimicrobial resistance genes to Pseudomonas aeruginosa
Source: BMC Genomics. 2017 Nov 10;18:859. doi: 10.1186/s12864-017-4216-2 (PMC5681832; doi:10.1186/s12864-017-4216-2)
Supplement: Supplementary file 5 — Overview of the closest related type strains based on the average nucleotide identity (ANI). (DOCX 16 kb) [file 12864_2017_4216_MOESM5_ESM.docx]

| **ID** | **WGS cluster** | **closest type strain (ANI)** |
| --- | --- | --- |
| P22 | 1 | *P. monteilii* (99,53) |
| P34 | 1 | *P. monteilii* (99,35) |
| E27 | 2 | *P. mosselii* (90,95) |
| P27 | 3 | *P. mosselii* (89,23) |
| P21B | 3 | *P. mosselii* (89,24) |
| P30 | 4A | *P. monteilii* (90,51) |
| P38 | 4B | *P. monteilii* (90,70) |
| P40 | 4B | *P. monteilii* (90,70) |
| P5 | 4B | *P. monteilii* (90,71) |
| P8 | 4B | *P. monteilii* (90,68) |
| P9 | 4B | *P. monteilii* (90,69) |
| P11 | 4B | *P. monteilii* (90,71) |
| P13 | 4B | *P. monteilii* (90,70) |
| P25 | 4B | *P. monteilii* (90,67) |
| E1 | 4B | *P. monteilii* (90,68) |
| E3 | 4B | *P. monteilii* (90,70) |
| E5 | 4B | *P. monteilii* (90,69) |
| E8 | 4B | *P. monteilii* (90,69) |
| E10 | 4B | *P. monteilii* (90,72) |
| E11 | 4B | *P. monteilii* (90,68) |
| E12 | 4B | *P. monteilii* (90,70) |
| E13 | 4B | *P. monteilii* (90,72) |
| E14 | 4B | *P. monteilii* (90,71) |
| E16 | 4B | *P. monteilii* (90,70) |
| E22 | 4B | *P. monteilii* (90,69) |
| P24 | 5A | *P. putida* (90,78) |
| P26 | 5A | *P. putida* (90,79) |
| P31 | 5A | *P. putida* (90,78) |
| P37 | 5A | *P. putida* (90,78) |
| P3 | 5A | *P. putida* (90,78) |
| P12 | 5A | *P. putida* (90,77) |
| P19 | 5A | *P. putida* (90,78) |
| P20 | 5A | *P. putida* (90,78) |
| P23 | 5A | *P. putida* (90,78) |
| E23 | 5A | *P. putida* (90,78) |
| P39 | 5A | *P. putida* (90,77) |
| P33 | 5B | *P. putida* (90,77) |
| P35 | 5B | *P. putida* (90,75) |
| P6 | 5B | *P. putida* (90,77) |
| P2 | 6A | *P. putida* (93,76) |
| E6 | 6A | *P. putida* (93,78) |
| E17 | 6A | *P. putida* (93,76) |
| E18 | 6A | *P. putida* (93,77) |
| E29 | 6A | *P. putida* (93,77) |
| P17 | 6B | *P. putida* (93,82) |
| P15 | 6C | *P. putida* (93,82) |
| P1 | 7 | *P. monteilii* (96,31) |
| P4 | 7 | *P. monteilii* (96,30) |
| P7 | 7 | *P. monteilii* (96,30) |
| P14 | 7 | *P. monteilii* (96,30) |
| P16 | 7 | *P. monteilii* (96,31) |
| P18 | 7 | *P. monteilii* (96,30) |
| P28 | 7 | *P. monteilii* (96,29) |
| P29 | 7 | *P. monteilii* (97,56) |
| P36 | 7 | *P. monteilii* (96,31) |
| P21A | 7 | *P. monteilii* (96,31) |
| E9 | 7 | *P. monteilii* (96,31) |
| P10 | 8 | *P. monteilii* (99,64) |
| P32 | 8 | *P. monteilii* (99,58) |

**Table S4.** **Overview of the closest related type strains based on the average nucleotide identity (ANI).**
