# Supplementary figures and images for: Genomic characterisation of clinical and environmental Pseudomonas putida group strains and determination of their role in the transfer of antimicrobial resistance genes to Pseudomonas aeruginosa
Source: BMC Genomics. 2017 Nov 10;18:859. doi: 10.1186/s12864-017-4216-2 (PMC5681832; doi:10.1186/s12864-017-4216-2)

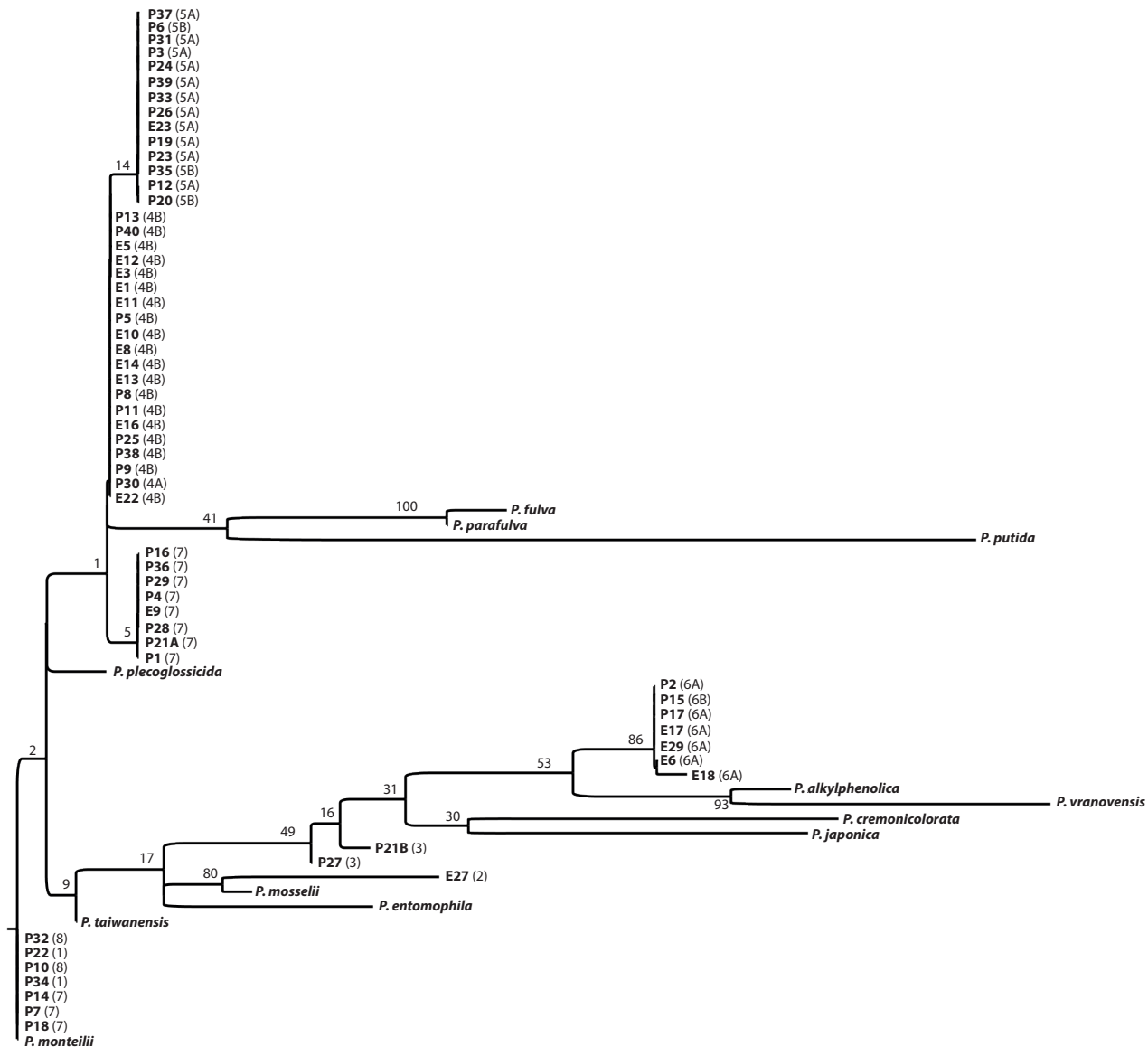

Supplement: Supplementary file 6 — 16S rRNA based maximum-likelihood tree of the P. putida group isolates and reference strains. The numbers displayed at the nodes are bootstrap values. The scale bar represents the expected number of changes per site. (PDF 456 kb) [file 12864_2017_4216_MOESM6_ESM.pdf]

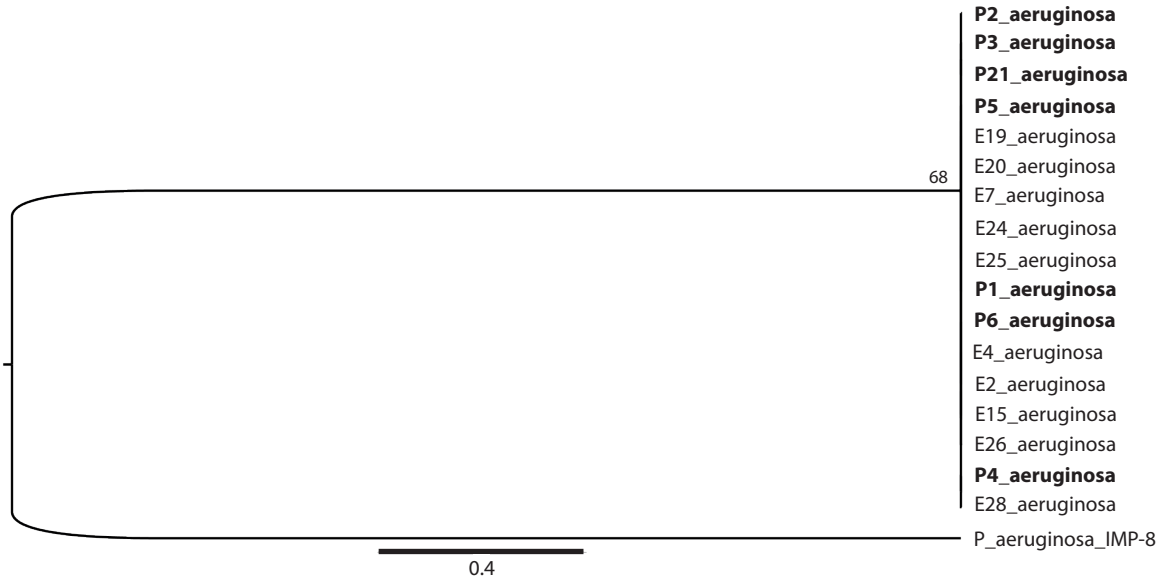

Supplement: Supplementary file 8 — Core genome maximum-likelihood phylogeny of 17 blaVIM-2 positive P. aeruginosa strains isolated from patients (n = 7, “P” as first letter) and water-related environmental sources (n = 10, “E” as first letter). All isolates are genetically highly similar. An outgroup strain (blaIMP-8 positive P. aeruginosa [29]) was introduced to ensure a proper visualisation of the strains’ sequence similarity and relatedness. The scale bar represents the expected number of changes per site. *P21 was also colonized with two different P. putida strains P21A and P21B. (PDF 295 kb) [file 12864_2017_4216_MOESM8_ESM.pdf]
