# Supplementary material for: Genomic characterisation of clinical and environmental Pseudomonas putida group strains and determination of their role in the transfer of antimicrobial resistance genes to Pseudomonas aeruginosa
Source: BMC Genomics. 2017 Nov 10;18:859. doi: 10.1186/s12864-017-4216-2 (PMC5681832; doi:10.1186/s12864-017-4216-2)
Supplement: Supplementary file 7 — Summary of epidemiological data of the P. putida isolates from the different NGS clusters. (DOCX 18 kb) [file 12864_2017_4216_MOESM7_ESM.docx]

| **Genetic pattern** | **Spatio-temporal link** | **Comment** | **Transmission probability** |
| --- | --- | --- | --- |
| **1** | P22 | no link | no evidence |
|  | P34 | no link | no evidence |
| **3** | P27/P21B | same ward same time | possible |
|  | P27/P21B | consecutive stay in same room (8 months later) | possible |
| **4A**  **4B** | P30  P5/ES | no link  E3, E13, E14 were isolated 3-5 weeks prior the stay of the patient in this room | no evidence  **probable** |
|  | P8 | no link | no evidence |
|  | P9 /P8 | consecutive stay in same room (8 days later) | **probable** |
|  | P11/P9 | consecutive stay in same room (1 days later) | **probable** |
|  | P13 | no link | no evidence |
|  | P25 | no link | no evidence |
|  | P38/P25 | consecutive stay in same room (6 month later) | possible |
|  | P38/ES | E10, E11 were isolated 23 months prior to the stay of the patient in this room | possible |
|  | P40 /P38 | consecutive stay in same room (14 days later) | **probable** |
|  | P40/ES | E3, E13, E14 were isolated 24 months prior the stay of the patient in this room | possible |
| **5A** | P3 | no link | no evidence |
|  | P12/ES | E23 was isolated 4 month prior to the stay of the patient in this room | possible |
|  | P19/P3 | consecutive stay in same room (11 months later) | possible |
|  | P19/P20/P24 | same ward same time | possible |
|  | P23/P12 | consecutive stay in same room (15 days later) | **probable** |
|  | P23/ES | E23 was isolated 13 month prior to the stay of the patient in this room | possible |
|  | P24/19 | consecutive stay in same room (15 days later) | **probable** |
|  | P26 | no link | no evidence |
|  | P31/P23 | consecutive stay in same room (5 months later) | possible |
|  | P31/ES | E23 was isolated 5 month prior to the stay of the patient in this room | possible |
|  | P37 | no link | no evidence |
|  | P39/P26 | consecutive stay in same room (8 months later) | possible |
| **5B** | P6 | no link | no evidence |
|  | P33 | no link | no evidence |
|  | P35 | no link | no evidence |
| **6A** | P2 /ES | E6, E17, E18 five weeks after the patient stayed in these two rooms | **probable** |
| **6B**  **6C** | P17  P15 | no link  no link | no evidence  no evidence |
| **7** | P1 | no link | no evidence |
|  | P4/ES | E9 was isolated from the patient room at the same time | **probable** |
|  | P7 | no link | no evidence |
|  | P14/P16 | same time same ward | possible |
|  | P16/P14 | consecutive stay in same room (4 days later) | **probable** |
|  | P18/P16 | consecutive stay in same room (6 days later) | **probable** |
|  | P21A/P4 | consecutive stay in same room (12 month later) | possible |
|  | P21A/ES | E9 was isolated 12 month prior to the stay of the patient in this room | possible |
|  | P28/P29 | same room same time | **probable** |
|  | P36 | no link | no evidence |
| **8** | P10 | no link | no evidence |
|  | P32/P10 | consecutive stay in same room (14 month later) | **probable** |

**Table S5. Summary of epidemiological data of the *P. putida* isolates from the different NGS clusters.** Spatio-temporal relationships between patients’ isolates and environmental isolates were determined. ES: environmental samples
