# Supplementary material for: Genomic characterisation of clinical and environmental Pseudomonas putida group strains and determination of their role in the transfer of antimicrobial resistance genes to Pseudomonas aeruginosa
Source: BMC Genomics. 2017 Nov 10;18:859. doi: 10.1186/s12864-017-4216-2 (PMC5681832; doi:10.1186/s12864-017-4216-2)
Supplement: Supplementary file 9 — Overview of predicted plasmid harbouring a bla VIM gene and the characteristics of the genetic bla VIM environment. (DOCX 31 kb) [file 12864_2017_4216_MOESM9_ESM.docx]

|  |  | **Predicted plasmids** | | **VIM environment based on WGS assembly** | |  |
| --- | --- | --- | --- | --- | --- | --- |
| **NGS Cluster** | **Strain ID** | **Result recycler** | **Result plasmidSPAdes** | **Genetic environment of *bla*_VIM_** | **Directly neighbouring ARGs** | **Presumed location of *bla*_VIM_** |
| 1 | P22 | no p-VIM | no p-VIM | Transposon elements  Integrase *IntI1*  Plasmid specific genes | *VIM-2, aacA4, aadA1, aph(3')-Ib* | indeterminate |
|  | P34 | no p-VIM | no p-VIM | Transposon elements  Integrase *IntI1*  Plasmid specific genes | *VIM-2, aacA4, aadA1, aph(3')-Ib* | indeterminate |
| 2 | E27 | no p-VIM | Size: 45338 bp  Transposon elements  pARGs:*aph(3')-XV, VIM-1, aacA4, aac(6')Ib-cr, aadA1, sul1* | Transposon elements | *aph(3')-XV, VIM-1, aacA4, aadA1, sul1* | plasmid |
| 3 | P21B | no p-VIM | no p-VIM | none | VIM-1 | chromosome |
|  | P27 | no p-VIM | Size: 46656  Transposon elements  *VIM-1, aacA4, aadA1, sul1* | Transposon elements | *VIM-1, aacA4, aadA1, sul1* | plasmid |
| 4A | P30 | no p-VIM | Size: 23239  Transposon elements  Integrase *IntI1*  Plasmid specific genes  pARGs: *VIM-1, aacA4, aadA1, sul1* | Transposon elements  Integrase *IntI1*  Plasmid specific genes | *VIM-1, aacA4, aadA1, sul1* | plasmid |
| 4B | P5 | no p-VIM | Size: 23068  Transposon elements  Integrase *IntI1*  pARGs: *VIM-1, aacA4, aph(3')-XV, aadA1, sul1* | Transposon elements  Integrase *IntI1*  Plasmid specific genes | *VIM-1, aacA4, aph(3')-XV, aadA1, sul1* | plasmid |
|  | P8 | no p-VIM | Size: 23068  Transposon elements  Integrase *IntI1*  pARGs: *VIM-1, aacA4, aph(3')-XV, aadA1, sul1* | Transposon elements  Integrase *IntI1* | *VIM-1, aacA4, aph(3')-XV, aadA1, sul1* | plasmid |
|  | P9 | no p-VIM | Size: 22429  Transposon elements  Integrase *IntI1*  pARGs: *VIM-1, aph(3')-XV, aadA1, sul1* | Transposon elements  Integrase *IntI1*  Plasmid specific genes | *VIM-1, aph(3')-XV, aadA1, sul1* | plasmid |
|  | P11 | no p-VIM | Size: 23068  Transposon elements  Integrase *IntI1*  pARGs: *VIM-1, aacA4, aph(3')-XV, aadA1, sul1* | Transposon elements  Integrase *IntI1*  Plasmid specific genes | *VIM-1, aacA4, aph(3')-XV, aadA1, sul1* | plasmid |
|  | P13 | no p-VIM | Size: 46993  Transposon elements  Integrase *IntI1*  pARGs: *VIM-1, aacA4, aadA1, sul1* | Transposon elements  Integrase *IntI1* | *VIM-1, aacA4, aadA1, sul1* | plasmid |
|  | P25 | no p-VIM | Size: 22429  Transposon elements  Integrase *IntI1*  pARGs: *VIM-1, aph(3')-XV, aadA1, sul1* | Transposon elements  Integrase *IntI1*  Plasmid specific genes | *VIM-1, aph(3')-XV, aadA1, sul1* | plasmid |
|  | P38 | no p-VIM | Size: 45742  Transposon elements  Integrase *IntI1*  Plasmid specific genes  pARGs: *VIM-1, aacA4, aadA1, sul1* | Transposon elements  Integrase *IntI1*  Plasmid specific genes | *VIM-1, aacA4, aadA1, sul1* | plasmid |
|  | P40 | no p-VIM | Size: 46354  Transposon elements  Integrase *IntI1*  Plasmid specific genes  pARGs: *VIM-1, aadA1, sul1* | Transposon elements  Integrase *IntI1* | *VIM-1, aadA1, sul1* | plasmid |
|  | E1 | Size: 23422  Transposon elements  Integrase *IntI1*  Plasmid specific genes  pARGs: *VIM-1, aacA4, aph(3')-XV, aadA1, sul1* | Size: 23068  pARGs: *VIM-1, aacA4, aph(3')-XV, aadA1, sul1*  Transposon elements  Integrase *IntI1* | Transposon elements  Integrase *IntI1*  Plasmid specific genes | *VIM-1, aacA4, aph(3')-XV, aadA1, sul1* | plasmid |
|  | E3 | Size: 23422 bp  Transposon elements  Integrase *IntI1*  Plasmid specific genes pARGs: *VIM-1, aacA4, aph(3')-XV, aadA1, sul1* | Size: 23068  Transposon elements  Integrase *IntI1*  pARGs: *VIM-1, aacA4, aph(3')-XV, aadA1, sul1* | Transposon elements  Integrase *IntI1*  Plasmid specific genes | *VIM-1, aacA4, aph(3')-XV, aadA1, sul1* | plasmid |
|  | E5 | no p-VIM | Size: 47905  pARGs: *VIM-1, aacA4, aph(3')-XV, aadA1, sul1*  Transposon elements  Integrase *IntI1* | Transposon elements  Integrase *IntI1* | *VIM-1, aacA4, aph(3')-XV, aadA1, sul1* | plasmid |
|  | E8 | no p-VIM | Size: 46993 Transposon elements  Integrase *IntI1*  pARGs: *VIM-1, aacA4, aadA1, sul1* | Transposon elements  Integrase *IntI1*  Plasmid specific genes | *VIM-1, aacA4, aadA1, sul1* | plasmid |
|  | E10 | Size: 1058 bp  none  pARGs: *VIM-1* | Size: 46993  Transposon elements  Integrase *IntI1*  pARGs: *VIM-1, aacA4, aadA1, sul1* | Transposon elements  Integrase *IntI1* | *VIM-1, aacA4, aadA1, sul1* | plasmid |
|  | E11 | no p-VIM | Size: 22429  Transposon elements  Integrase *IntI1*  pARGs: *VIM-1, aph(3')-XV, aadA1, sul1* | Transposon elements  Integrase *IntI1*  Plasmid specific genes | *VIM-1, aph(3')-XV, aadA1, sul1* | plasmid |
|  | E12 | no p-VIM | Size: 46993  Transposon elements  Integrase *IntI1*  pARGs: *VIM-1, aacA4, aadA1, sul1* | Transposon elements  Integrase *IntI1*  Plasmid specific genes | *VIM-1, aacA4, aadA1, sul1* | plasmid |
|  | E13 | no p-VIM | Size: 23068  Transposon elements  Integrase *IntI1*  pARGs: *VIM-1, aacA4, aph(3')-XV, aadA1, sul1* | Transposon elements  Integrase *IntI1*  Plasmid specific genes | *VIM-1, aacA4, aph(3')-XV, aadA1, sul1* | plasmid |
|  | E14 | no p-VIM | Size: 21517  Transposon elements  Integrase *IntI1*  pARGs: *VIM-1, aadA1, sul1* | Transposon elements  Integrase *IntI1*  Plasmid specific genes | *VIM-1, aadA1, sul1* | plasmid |
|  | E16 | no p-VIM | Size: 47266  Transposon elements  Integrase *IntI1*  pARGs: *VIM-1, aph(3')-XV, aadA1, sul1* | Transposon elements  Integrase *IntI1*  Plasmid specific genes | *VIM-1, aph(3')-XV, aadA1, sul1* | plamid |
|  | E22 | no p-VIM | Size: 43995  Transposon elements  Integrase *IntI1*  pARGs: *VIM-1, aph(3')-XV, aadA1, sul1* | Transposon elements  Integrase *IntI1* | *VIM-1, aph(3')-XV, aadA1, sul1* | plasmid |
| 5A | P3 | no p-VIM | no p-VIM | VIM-1: Transposon elements  Integrase *IntI1*  VIM-2:none | 1. *VIM-1, aacA4, sul1*   2. *VIM-2, aph(3')-Ib* | chromosome |
|  | P12 | no p-VIM | no p-VIM | Transposon elements  Integrase *IntI1* | *VIM-1, aacA4, sul1* | chromosome |
|  | P19 | no p-VIM | no p-VIM | VIM-1: none  VIM-2: Transposon elements  Integrase *IntI1*  Plasmid specific genes | *1.VIM-1,*  2. *VIM-2, aadA1, aph(3')-Ib, aacA4,* | indeterminate |
|  | P20 | no p-VIM | no p-VIM | Transposon elements  Integrase *IntI1* | *VIM-1, aacA4, sul1* | chromosome |
|  | P23 | no p-VIM | no p-VIM | Transposon elements  Integrase *IntI1* | *VIM-1, aacA4, sul1* | chromosome |
|  | P24 | no p-VIM | no p-VIM | VIM-1: Transposon elements  VIM-2: none | *1.VIM-1, aacA4, sul1*  2. *VIM-2* | chromosome |
|  | P26 | no p-VIM | no p-VIM | VIM-1: none  VIM-2: none | *1.VIM-1,*  2. *VIM-2* | chromosome |
|  | P31 | no p-VIM | no p-VIM | Transposon elements  Integrase *IntI1* | *VIM-1, aacA4, sul1* | chromosome |
|  | P37 | no p-VIM | no p-VIM | Transposon elements  Integrase *IntI1* | *VIM-1, aacA4, sul1* | chromosome |
|  | P39 | no p-VIM | no p-VIM | VIM-1: none  VIM-2: Transposon elements  Plasmid specific genes | *1.VIM-1,*  2. *VIM-2, aadA1, aph(3')-Ib, aacA4* | indeterminate |
|  | E23 | no p-VIM | no p-VIM | Transposon elements  Integrase *IntI1* | *VIM-1, aacA4, sul1* | chromosome |
| 5B | P6 | no p-VIM | no p-VIM | Transposon elements  Integrase *IntI1*  Plasmid specific genes | *VIM-2, aacA4, aadA1, aph(3')-Ib* | indeterminate |
|  | P33 | no p-VIM | no p-VIM | Transposon elements  Integrase *IntI1*  Plasmid specific genes | *VIM-2, aacA4, aadA1, aph(3')-Ib* | indeterminate |
|  | P35 | no p-VIM | no p-VIM | Transposon elements  Integrase *IntI1*  Plasmid specific genes | *VIM-2, aacA4, aadA1, aph(3')-Ib* | indeterminate |
| 6A | P2 | no p-VIM | no p-VIM | none | *VIM-2* | chromosome |
|  | E6 | no p-VIM | no p-VIM | none | *VIM-2* | chromosome |
|  | E17 | no p-VIM | no p-VIM | none | *VIM-2* | chromosome |
|  | E18 | no p-VIM | no p-VIM | Transposon elements  Integrase *IntI1*  Plasmid specific genes | *VIM-2, aacA4, aadA1, aph(3')-Ib* | indeterminate |
|  | E29 | no p-VIM | no p-VIM | Transposon elements  Integrase *IntI1*  Plasmid specific genes | *VIM-2, aacA4, aadA1, aph(3')-Ib* | indeterminate |
| 6B | P17 | no p-VIM | Size: 79160  Transposon elements  Integrase *IntI1*  pARGs: *VIM-1, aacA4, aph(3')-XV, aadA1, sul1, strB* | Transposon elements  Integrase *IntI1* | *VIM-1, aacA4, aph(3')-XV, aadA1, sul1* | plasmid |
| 6C | P15 | no p-VIM | no p-VIM | Transposon elements  Integrase *IntI1*  Plasmid specific genes | *VIM-2, aacA4, aadA1, aph(3')-Ib* | indeterminate |
| 7 | P1 | no p-VIM | no p-VIM | Transposon elements  Integrase *IntI1*  Plasmid specific genes | *VIM-2, aacA4, aadA1, aph(3')-Ib* | indeterminate |
|  | P4 | no p-VIM | Size: 96214  Transposon elements  Integrase *IntI1*  Plasmid specific genes  pARGs: *VIM-2, aacA4, aadA1, aph(3')-Ib* | Transposon elements  Integrase *IntI1*  Plasmid specific genes | *VIM-2, aacA4, aadA1, aph(3')-Ib* | plasmid |
|  | P7 | no p-VIM | no p-VIM | Transposon elements  Integrase *IntI1*  Plasmid specific genes | *VIM-2, aacA4, aadA1, aph(3')-Ib* | indeterminate |
|  | P14 | no p-VIM | no p-VIM | Transposon elements  Integrase *IntI1*  Plasmid specific genes | *VIM-2, aacA4, aadA1, aph(3')-Ib* | indeterminate |
|  | P16 | no p-VIM | no p-VIM | Transposon elements  Integrase *IntI1*  Plasmid specific genes | *VIM-2, aacA4, aadA1, aph(3')-Ib* | indeterminate |
|  | P18 | no p-VIM | no p-VIM | Transposon elements  Integrase *IntI1*  Plasmid specific genes | *VIM-2, aacA4, aadA1, aph(3')-Ib* | indeterminate |
|  | P21A | no p-VIM | no p-VIM | Transposon elements  Integrase *IntI1*  Plasmid specific genes | *VIM-2, aacA4, aadA1, aph(3')-Ib* | indeterminate |
|  | P28 | no p-VIM | no p-VIM | Transposon elements  Integrase *IntI1*  Plasmid specific genes | *VIM-2, aacA4, aadA1, aph(3')-Ib* | indeterminate |
|  | P29 | no p-VIM | no p-VIM | Transposon elements  Integrase *IntI1*  Plasmid specific genes | *VIM-2, aacA4, aadA1, aph(3')-Ib* | indeterminate |
|  | P36 | no p-VIM | no p-VIM | Transposon elements  Integrase *IntI1*  Plasmid specific genes | *aph(3')-Ib, aadA1, aac(6')Ib-cr, aacA4, VIM-2* | indeterminate |
|  | E9 | no p-VIM | no p-VIM | Transposon elements  Integrase *IntI1*  Plasmid specific genes | *VIM-2, aacA4, aadA1, aph(3')-Ib* | indeterminate |
| 8 | P10 | no p-VIM | Size: 19563  Transposon elements  Integrase *IntI1*  Plasmid specific genes  pARGs: *VIM-1, aacA4, aadA1, sul1* | Transposon elements  Integrase *IntI1*  Plasmid specific genes | *VIM-1, aacA4, aadA1, sul1* | plasmid |
|  | P32 | no p-VIM | Size: 48596  Transposon elements  Integrase *IntI1*  pARGs: *VIM-1, aacA4, aadA1, sul1* | Transposon elements  Integrase *IntI1* | *VIM-1, aacA4, aadA1, sul1* | plasmid |
| **PA** | P1_aer | no p-VIM | no p-VIM | none | *VIM-2* | chromosome |
|  | P2_aer | no p-VIM | no p-VIM | none | *VIM-2* | chromosome |
|  | P3_aer | no p-VIM | no p-VIM | Transposon elements  Integrase *IntI1* | *VIM-2, dfrB5, aac(3)-Id* | chromosome |
|  | P4_aer | no p-VIM | no p-VIM | Transposon elements  Integrase *IntI1* | *VIM-2, dfrB5, aac(3)-Id* | chromosome |
|  | P5_aer | no p-VIM | no p-VIM | Transposon elements  Integrase *IntI1* | *VIM-2, dfrB5, aac(3)-Id* | chromosome |
|  | P6_aer | no p-VIM | no p-VIM | Transposon elements  Integrase *IntI1* | *VIM-2, dfrB5, aac(3)-Id* | chromosome |
|  | P21 | no p-VIM | no p-VIM | Transposon elements  Integrase *IntI1* | *dfrB5, VIM-2* | chromosome |
|  | E2 | no p-VIM | no p-VIM | none | *VIM-2* | chromosome |
|  | E4 | no p-VIM | no p-VIM | none | *VIM-2* | chromosome |
|  | E7 | no p-VIM | no p-VIM | Transposon elements  Integrase *IntI1* | *VIM-2, dfrB5, aac(3)-Id* | chromosome |
|  | E15 | no p-VIM | no p-VIM | Transposon elements  Integrase *IntI1* | *VIM-2, dfrB5, aac(3)-Id* | chromosome |
|  | E19 | no p-VIM | no p-VIM | none | *VIM-2* | chromosome |
|  | E20 | no p-VIM | no p-VIM | none | *VIM-2* | chromosome |
|  | E24 | no p-VIM | no p-VIM | none | *VIM-2* | chromosome |
|  | E25 | no p-VIM | no p-VIM | Transposon elements  Integrase *IntI1* | *VIM-2, dfrB5, aac(3)-Id* | chromosome |
|  | E26 | no p-VIM | no p-VIM | Transposon elements  Integrase *IntI1* | *VIM-2, dfrB5* | chromosome |
|  | E28 | no p-VIM | no p-VIM | Transposon elements  Integrase *IntI1* | *aac(3)-Id, dfrB5, VIM-2* | chromosome |

**Table S6.** Overview of predicted plasmid harbouring a *bla*_VIM_ gene and the characteristics of the genetic *bla*_VIM_ environment. No plasmids harbouring a *bla*_VIM_ gene were predicted in the *P. aeruginosa* isolates and no plasmid specific genes were detected in the *bla*_VIM_ flanking region based on WGS assembly data and annotation. Plasmids containing *bla*_VIM_ were predicted in three *P. putida* strains by applying Recyler and in 24 *P. putida* strains by applying plasmidSPAdes. PlasmidFinder identified no plasmids in our WGS datasets.

In order to assess the genetic location of the *bla*_VIM_ gene, we presumed a plasmid location most likely if a plasmid was predicted by one of the plasmid prediction tools. The location of the *bla*_VIM_ gene was considered indeterminate if no plasmid was predicted, but plasmid specific genes were identified in close proximity to the *bla*_VIM_ gene on the contig of the assembled WGS dataset. Chromosomal localisation was considered most likely if no plasmids were predicted and no plasmid specific genes could be detected on the *bla*_VIM_ harbouring contig.
